# Supplementary material for: Circular extrachromosomal DNA promotes tumor heterogeneity in high-risk medulloblastoma
Source: Nat Genet. 2023 Nov 9;55(12):2189–99. doi: 10.1038/s41588-023-01551-3 (PMC10703696; doi:10.1038/s41588-023-01551-3)
Supplement: Supplementary file 1 — Supplementary Notes and Figures. [file 41588_2023_1551_MOESM1_ESM.pdf]

nature genetics

Article https://doi.org/10.1038/s41588-023-01551-3

Circular extrachromosomal DNA promotes tumor heterogeneity in high-risk medulloblastoma

In the format provided by the authors and unedited

SUPPLEMENTARY MATERIAL

Supplementary Note 1: Classical mediation analyses link ecDNA to known predictors of MB survival.

We hypothesized that the established effect of *TP53* mutation on patient survival[1] may be mediated, at least partially, by ecDNA **(Extended Data Fig. 3a)**. To test this, we conducted a mediation analysis using the Baron Kenny approach[2]. Briefly, variable *M* (ie, ecDNA status) is considered a mediator of the effect of independent variable *X* (ie, *TP53* mutation) on outcome *Y* (ie, patient survival) if (1) *X* significantly predicts *Y*; (2) *X* significantly predicts *M*; and (3) in a multiple regression of *Y* on *X* and *M*, *M* significantly predicts *Y* and the effect of *X* on *Y* is reduced relative to (1)[3]. To test (1), we fitted a log-normal accelerated failure time (AFT) regression of PFS parameterized on age, sex, molecular subgroup and *TP53* mutation status, using maximum likelihood estimation (MLE). Without information on ecDNA, this model estimates that *TP53* mutation significantly reduces survival time by 77% (μ = -1.46, p < 0.005, Wald test; **Extended Data Fig. 3b**). For (2), we have already shown that *TP53* mutation strongly predicts the presence of ecDNA, given molecular subgroup. To test (3), we refitted our AFT model parameterized on age, sex, molecular subgroup, p53 mutation, and ecDNA status. This model estimates that ecDNA significantly reduces survival time by 56% (μ = -0.81, p < 0.005; **Extended Data Fig. 3c**). The same model estimates an insignificant reduction in survival time for *TP53* mutation, with a coefficient of lesser magnitude than that of (1) (μ = -0.94, p = 0.06). We therefore concluded that the known prognostic effect of *TP53* mutation on PFS in medulloblastoma patients is partially mediated by ecDNA amplification.

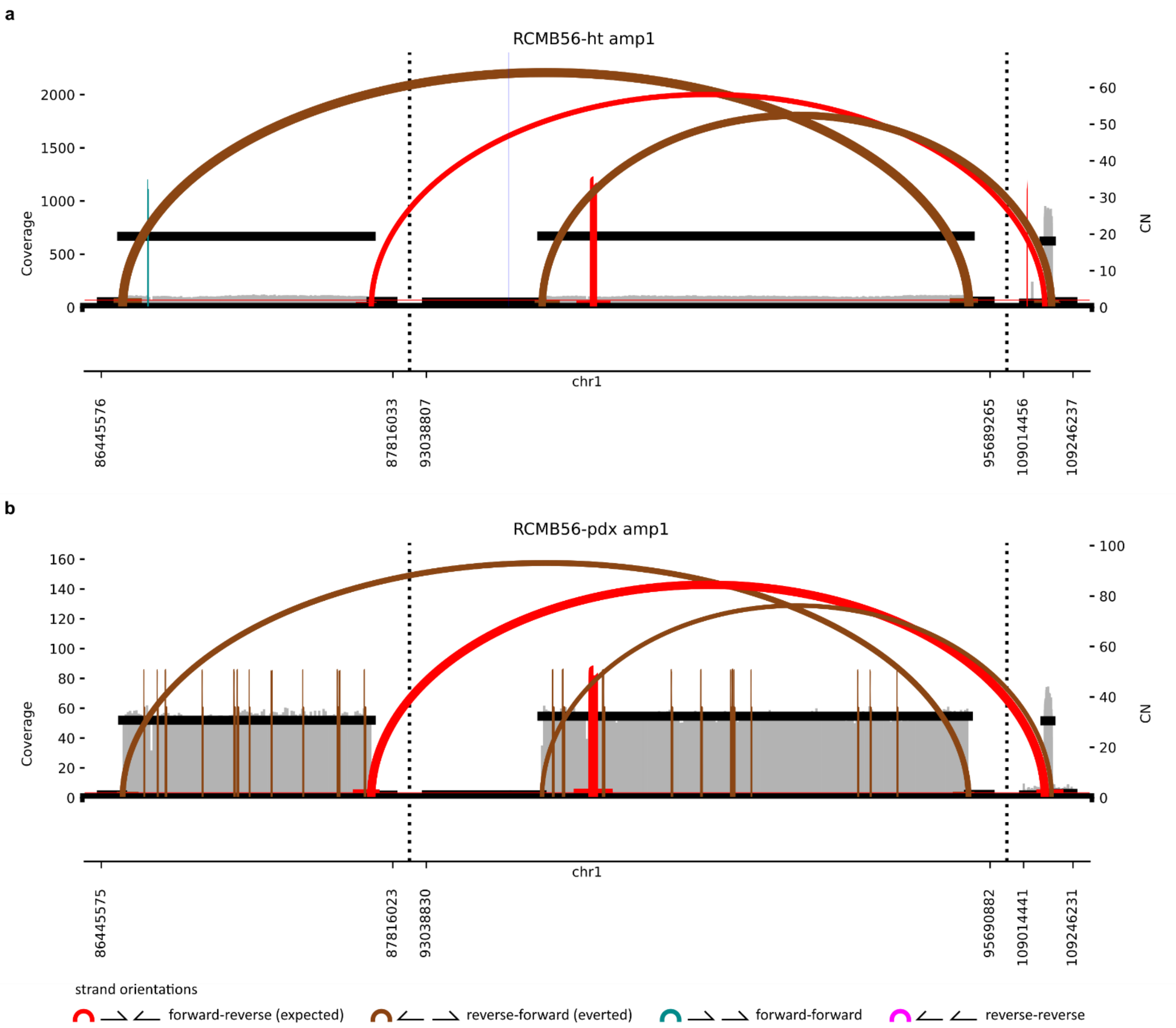

Supplementary Figure 1: AmpliconArchitect reconstructions of the ecDNA amp1 from RCMB56 samples. (**a**) AA reconstruction of amp1 from RCMB56-ht (primary tumor) WGS, with colored arcs representing discordant paired-end breakpoints. Arc thickness indicates the number of discordant reads supporting that junction. (**b**) The same for lcWGS of RCMB56-pdx.

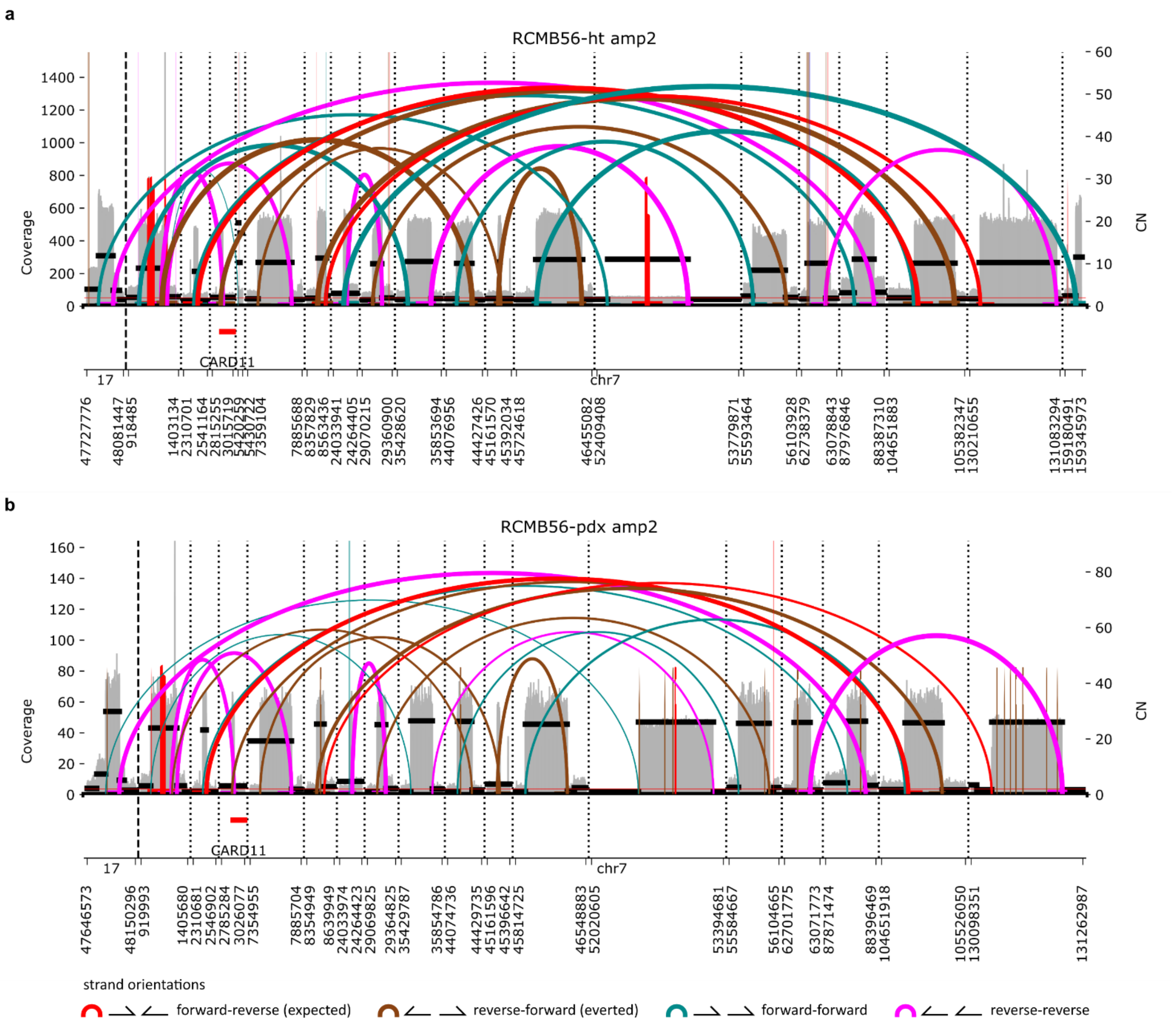

Supplementary Figure 2: AmpliconArchitect reconstructions of the ecDNA amp2 from RCMB56 samples. (**a**) AA reconstruction of amp2 from RCMB56-ht (primary tumor) WGS, with colored arcs representing discordant paired-end breakpoints. Arc thickness indicates the number of discordant reads supporting that junction. (**b**) The same for lcWGS of RCMB56-pdx.

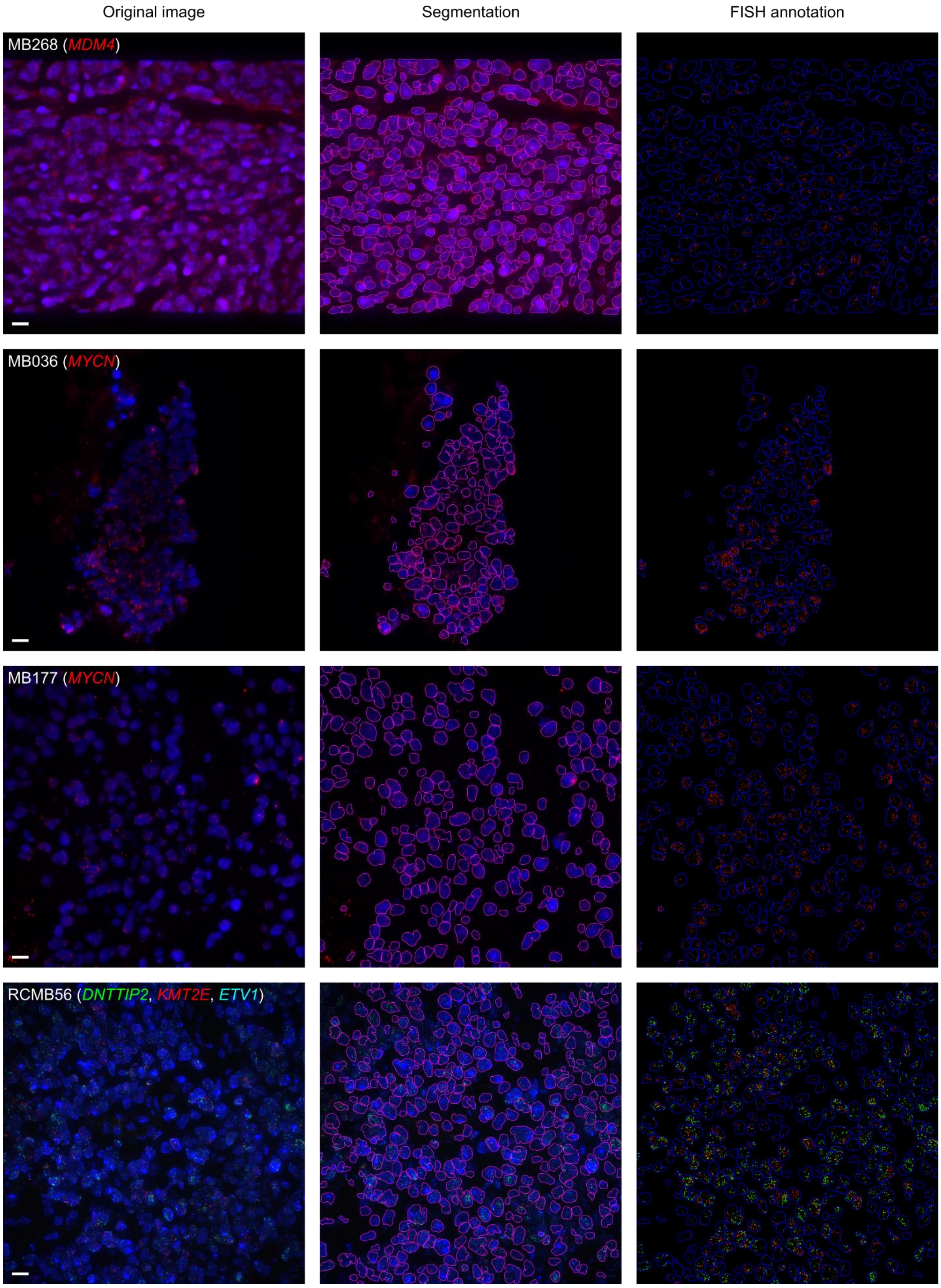

Supplementary Figure 3: Automated segmentation and annotation of gene copy number in interphase FISH imaging. Example annotations of FISH imaging of human MB FFPE tissue sections. Left column: merged images of DAPI channel (blue) and FISH probes (red: *MDM4*, *MYCN*, *KMT2E*; green: *DNTTIP2*; aqua: *ETV1*). Center column: automated nuclear segmentations annotated by NuSeT[4]. Right column: FISH spots indicating ecDNA as annotated by ecSeg-i[5]. Each image is representative of series of 18 (MB268), 13 (MB036), 11 (MB177), and 45 (RCMB56-ht) images. All scale bars 10μm.

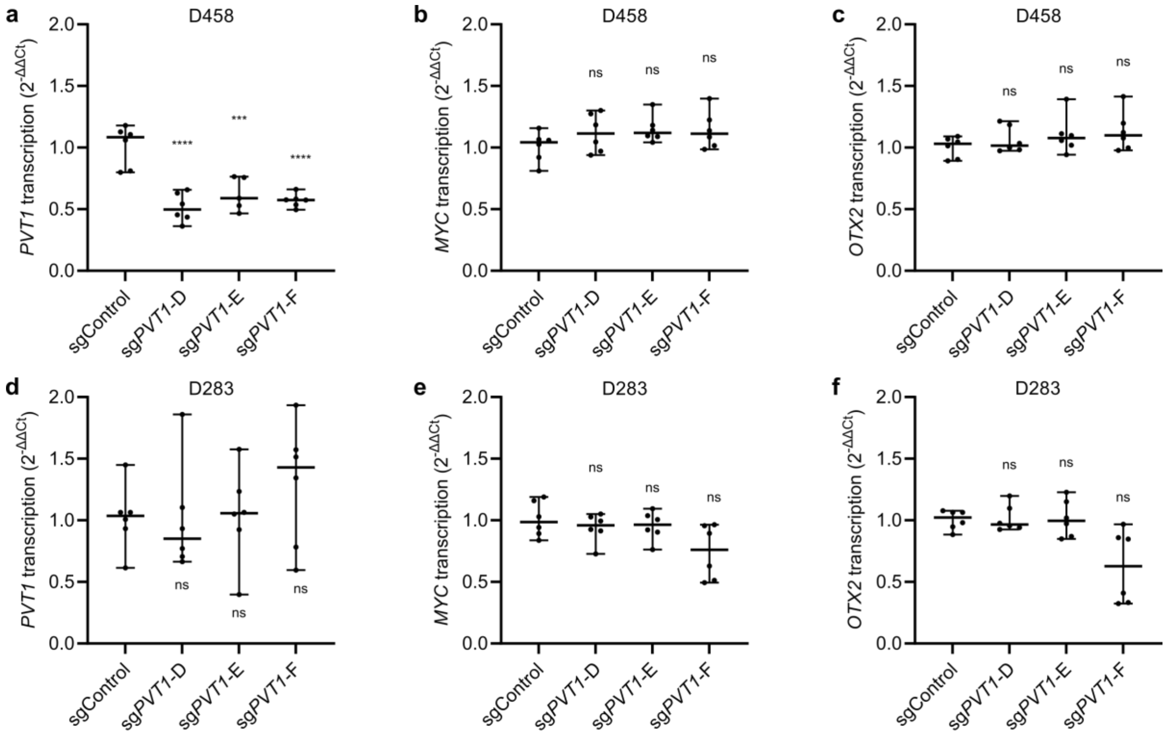

Supplementary Figure 4: *PVT1* promotes D458 cell proliferation. Relative expression of *PVT1* (**a-b**), *MYC* (**c-d**), and *OTX2* (**e-f**) measured by qPCR ($2^{-\Delta\Delta Ct}$), in D458 and D283 cell lines upon CRISPRi targeting of the *PVT1* promoter which promotes D458 proliferation (see also **Fig. 4h**). sgNT: nontargeting control; sg*PVT1*-D-F: sgRNAs targeting the *PVT1* promoter at D458_peak_30920, positions chr8:127794266, chr8:127794773, and chr8:127794945 respectively (hg38). qPCR was performed on all guides in triplicate; each technical replicate is shown. Bars represent median +/- 95% CI. *** $p = 0.0002$; **** $p < 0.0001$; one-sided nested ANOVA with Dunnett's correction.

Supplementary Note 2

Detail on WGS processing pipelines

CBTN – Children's Brain Tumor Network (114 biosamples from 101 patients). WGS of medulloblastoma tumor biopsies were identified using the Gabriella Miller KidsFirst Data Resource center portal (https://portal.kidsfirstdrc.org/) on 29 May 2020. Patients were originally sequenced as part of the Pediatric Brain Tumor Atlas (PBTA) (https://cbtn.org/pediatric-brain-tumor-atlas). WGS data were preprocessed and aligned using the KidsFirst harmonized WGS pipeline (hg38) and subsequently analyzed using Cavatica (https://cavatica.sbgenomics.com/), on the cloud genomic analysis platform for KidsFirst genomic data. Docker containers containing fingerprint and AmpliconArchitect software were installed on the Cavatica cloud genomics platform (see Methods: "EcDNA detection and classification"). As described in Lilly *et al.*, 2022, patients are consented by one of 32 participating sites and enrolled on a local IRB-approved protocol which includes key language to enable prospective collection of, future research on, and sharing of, de-identified surgical specimens, patient demographics, medical history, diagnoses, treatments, and clinical imaging. CHOP reviews each site's regulatory documents prior to submission and maintains copies along with annual approvals[6].

St Jude (79 patients). WGS of MB biopsies were identified using the St Jude Cloud Data Portal (https://platform.stjude.cloud/data/) on 11 February 2020. WGS was preprocessed and aligned by bwa-mem to hg38 as described in McLeod *et al*[7]. Docker containers of fingerprint and AmpliconArchitect software were installed on the DNANexus cloud genomics platform (see Methods: "EcDNA detection and classification"). Validation of patient consent protocol availability (including active monitoring for revocation of previous consent) and sample de-identification are implemented in the data processing pipeline at St. Jude Cloud in accordance with legal and ethical guidelines as described in McLeod *et al*., 2021[7].

ICGC – International Cancer Genome Consortium (237 patients). WGS of MB tumor biopsies was identified using the ICGC Data Portal (https://dcc.icgc.org/) on 12 May 2020 and downloaded from the Collaboratory cloud genomics platform using the ICGC Score download client 5.0.0. These tumor genomes were previously sequenced, aligned and deposited with ICGC in other publications (GRCh37)[8,9]. All patient data was collected after receiving informed consent according to ICGC guidelines and as approved by the institutional review boards of contributing centers[8].

Archer et al. (43 patients). Primary medulloblastoma patient samples, including FFPE slides, were obtained with informed consent according to the International Cancer Genome Consortium (ICGC) guidelines as approved by the Ethics Committee of the Medical Faculty at Heidelberg University, and as approved by the institutional review board of contributing center Nikolay Nilovich Burdenko Neurosurgical Institute in Moscow[8]. All samples were de-identified. Sequencing data were aligned to human genome reference GRCh37 processed according to the best practice pipelines at the Cancer Genome Analysis group at the Broad Institute[10].

Rady Children's Hospital (8 patients). Tumor biopsies were collected and consented for research as part of the Rady Molecular Tumor Board (MTB). Paired end reads were acquired from Rady's Children hospital. Sequencing depth for all samples were at least 30x. Raw fastq reads were aligned to UCSC hg38 coordinates using BWA v0.7.17-r1188[11]. Reads were sorted by samtools v0.1.19[12], marked for duplicates with Picard Tools v2.12.3, and recalibrated with GATK v3.8-1-0[13-15].

Cell lines and PDX models (D458 and RCMB56). WGS was performed at the UCSD IGM Genomics and Sequencing Core (hg38) for all samples except D283, for which no WGS was obtained. Preprocessing and alignment was performed using the same protocol as the RCH patient biopsies above.

Supplementary Note 3

Detail on ATAC-seq of tumor and cell line samples

Archer et al. samples. ATAC-seq was performed at the Massachusetts Institute of Technology (Cambridge, MA). At least 25mg of pulverized frozen tissue from samples previously included in proteomics analysis[10] were sequenced by ATAC-seq according to an established protocol for bulk ATAC-seq of frozen neuronal cells[16]. Sequencing was performed on the Illumina HiSeq 2000 platform. Samples included in subsequent analyses passed the following quality control thresholds: PCR bottleneck coefficient 1 (PBC1) > 0.7, PCR bottleneck coefficient 2 (PBC2) > 3, non-redundant read fraction (NRF) > 0.8 and TSS enrichment > 2.6. These samples had at least 13 million uniquely mapped single-end reads (GRCh38). Sequencing quality was evaluated by FastQC v0.11.7.

RCMB56 and D458. ATAC-seq was performed on frozen dissociated cells at ActiveMotif, Inc. (San Diego, CA). Briefly, ~100K cells were permeabilized and transposase loaded with sequencing adapters was added. Sequencing was performed on the Illumina NextSeq 6000 platform. Samples had PBC1 > 0.9, PBC2 > 20, NRF > 0.5 and at least 30 million uniquely mapped paired-end reads (hg38).

ATAC-seq data processing. Following ENCODE best practices, ATAC-seq reads were trimmed using trimmomatic 0.36[17]; quality-checked using fastqc 0.11.7; aligned using Bowtie 2.3.4.3[18]; indexed using Samtools 1.10[12]; and deduplicated using Picard 2.20.8. ATAC-seq peaks were called using MACS2 2.1.2[19] at BH-corrected threshold $q < 0.05$.

Supplementary Note 4

Detail on Hi-C of tumor and cell line samples

Archer et al. samples (except MB248). Hi-C on frozen tumor tissue sample was carried out according to an existing protocol[20]. In brief, frozen tissues were pulverized using a mortar and pestle kept cold on a bed of dry ice into a fine powder. Tissue powder was then suspended in 5mL DPBS, fixed with 2% formaldehyde for 10 minutes, and quenched by addition of 0.2M Glycine. Fixed tissue was pelleted by centrifugation, washed 1x with DPBS, and then flash frozen until ready for further processing. For Hi-C experiments, fixed frozen tissue pellets were resuspended in 3mL of lysis buffer (10mM Tris-HCl pH 8.0, 5mM $CaCl_2$, 3mM MgAc, 2mM EDTA, 0.2mM EGTA, 1mM DTT, 0.1mM PMSF, 1X Complete Protease Inhibitors). Sample was transferred to an M-tube and dissociated using a GentleMACS Tissue dissociator (Miltenyi) using the "Protein M-tube" setting. The sample was removed from the M-tube into a 50mL conical. The M-tube was washed with 3mL lysis buffer with 0.4% Triton X-100, and this wash was combined with the original 3mLs of sample for a total volume of 6mLs and final concentration of 0.2% Triton X-100. The sample was then passed through a 40µM cell strainer. The strainer was washed with an additional 2mLs of lysis buffer with 0.2% Triton X-100. The sample was then centrifuged and washed with 1mL of lysis buffer with 0.2% Triton X-100. After centrifugation, the sample was resuspended in 0.5% SDS and processed with the *in situ* Hi-C method[21] using the MboI enzyme. Libraries were prepared using the Illumina TruSeq LT sequencing adapters. Initial QC sequencing was performed on a MiSeq to assess library quality, and if sufficient, was subject to production scale sequencing on the HiSeq X or NovaSeq platform.

D458, *RCMB56*, and *MB248*. Hi-C experiments were carried out by Arima Genomics, Inc (San Diego, CA) using the Arima-HiC Kit protocol with MboI restriction enzyme. Subsequently, Illumina-compatible sequencing libraries were prepared by shearing the proximally ligated DNA and then size-selecting DNA fragments using SPRI beads. The size-selected fragments containing ligation junctions were enriched using Enrichment Beads (provided in the Arima-HiC Kit), and converted into Illumina-compatible sequencing libraries using the Swift Accel-NGS 2S Plus kit (#21024) reagents. After adapter ligation, DNA was PCR amplified and purified using SPRI beads. Purified DNA underwent standard QC (qPCR and Bioanalyzer) and sequenced on the NovaSeq platform.

Supplementary Note 5

Detail on pooled CRISPRi proliferation screen

sgRNAs targeting the D458 ecDNA regulome were designed using CHOPCHOP v3[22]. All sgRNAs with no exact homology elsewhere in the genome (ie, mm0 = 0) were included, totaling 32,530 sgRNAs (**Supplementary Table 23**). Targeted regions included 645 accessible regions on the D458 ecDNA, and putative enhancers of MYC in medulloblastoma[23]. As positive controls, sgRNAs targeting the promoter regions of *POLR2A*, *MYC* and *OTX2* were also included. No non-targeting sgRNAs were included; however 4 on chr8 with no evidence of regulatory activity in D458 were also included. Pooled oligonucleotides were generated at Genewiz, Inc. The library was amplified and packaged into lentivirus in 293FT cells (Invitrogen #R70007) by SBP Functional Genomics Core Facility.

D283 Med and D458 Med cells were infected by the lentivirally-packaged sgRNA library at a MOI of 0.3 at a multiplicity of 1000 cells/sgRNAs. Two independent infections were performed for each cell line as biological replicates (A and B). Forty-eight hours post-infection, infected cells were selected using puromycin (1 µg/ml). After 2 days selection, the MOI was measured and 30 million of cells were collected for NGS sequencing (timepoint 0 “T0”) as a reference baseline for sgRNA distribution. Another 30 million cells were re-plated into culture flasks and maintained for 21 days (timepoint 21 “T21”).

To ensure sgRNA representation in each replicate, 30 million cells were maintained throughout the screen during cell passage every 3-4 days. Cells were harvested at T21, and genomic DNA extraction was performed using the NucleoSpin Blood XL kit. The NGS samples were prepared by the SBP Functional Genomics Core Facility using two-step PCR amplification of the integrated sgRNA using the genomic DNA as a template. For the first PCR, “PCR1” sgRNAs were amplified using Herculase II Fusion DNA Polymerases (Agilent #600675) from total genomic DNA isolated from each replicate at T0 and T21. 4ug of genomic DNA was used for each 50ul reaction. PCR1 products were cleaned using QIAquick PCR Purification Kit (Qiagen). For the second PCR “PCR2”, 5µl of each cleaned PCR1 product was used for each 50ul reaction with primers designed to include Illumina Adaptors and unique barcodes to allow for multiplexed NGS. PCR2 products were bead-purified (AMPure XP, Beckman), and eluted in 30µl of low-EDTA TE buffer. Final concentrations of the desired products were determined by TapeStation (Agilent) and equimolar amounts from each sample were pooled for sequencing.

Following selection, libraries were sequenced to a minimum depth of 60M reads per sample. Sequencing data QC, alignment and preprocessing was performed using MAGeCK-VISPR v0.4.1653[24]. At least 85% of reads per sample were uniquely mappable to a sgRNA sequence, and fewer than 100 sgRNAs (0.3%) were absent from any library. CRISPRi score tracks were generated using CRISPR-SURF v1.0[25]. To identify D458-specific functional loci, T21 and T0 for both cell lines were compared using the Robust Rank Aggregation (RRA) algorithm of MAGeCK. Essential loci were identified for each cell line where guides mapping to that locus were depleted at T21 (corrected p-value < 0.05). Independently, D458-specific loci were identified by a linear model parameterized on cell line and timepoint (MAGeCK MLE, corrected p-value < 0.05). The final set of D458-specific functional loci was the intersection of the loci identified by these two analyses. Genomic tracks were plotted in IGV desktop v2.9.2[26].

SUPPLEMENTARY MATERIAL REFERENCES

1 Zhukova, N. *et al.* Subgroup-Specific Prognostic Implications of TP53 Mutation in Medulloblastoma. *Journal of Clinical Oncology* **31**, 2927-2935, doi:10.1200/JCO.2012.48.5052 (2013).

2 Baron, R. M. & Kenny, D. A. The moderator–mediator variable distinction in social psychological research: Conceptual, strategic, and statistical considerations. *Journal of Personality and Social Psychology* **51**, 1173-1182, doi:10.1037/0022-3514.51.6.1173 (1986).

3 Jung, S. Y. *et al.* Comorbidity as a Mediator of Survival Disparity Between Younger and Older Women Diagnosed With Metastatic Breast Cancer. *Hypertension* **59**, 205-211, doi:10.1161/HYPERTENSIONAHA.111.171736 (2012).

4 Yang, L. *et al.* NuSeT: A deep learning tool for reliably separating and analyzing crowded cells. *PLOS Computational Biology* **16**, e1008193, doi:10.1371/journal.pcbi.1008193 (2020).

5 Rajkumar, U. *et al.* EcSeg: Semantic Segmentation of Metaphase Images Containing Extrachromosomal DNA. *iScience* **21**, 428-435, doi:10.1016/j.isci.2019.10.035 (2019).

6 Lilly, J. V. *et al.* The children's brain tumor network (CBTN) - Accelerating research in pediatric central nervous system tumors through collaboration and open science. *Neoplasia (New York, N.Y.)* **35**, 100846, doi:https://doi.org/10.1016/j.neo.2022.100846 (2023).

7 McLeod, C. *et al.* St. Jude Cloud-a Pediatric Cancer Genomic Data Sharing Ecosystem. *Cancer discovery*, CD-20-1230, doi:10.1158/2159-8290.CD-20-1230 (2021).

8 Northcott, P. A. *et al.* The whole-genome landscape of medulloblastoma subtypes. *Nature* **547**, 311-317, doi:10.1038/nature22973 (2017).

9 Campbell, P. J. *et al.* Pan-cancer analysis of whole genomes. *Nature* **578**, 82-93, doi:10.1038/s41586-020-1969-6 (2020).

10 Archer, T. C. *et al.* Proteomics, Post-translational Modifications, and Integrative Analyses Reveal Molecular Heterogeneity within Medulloblastoma Subgroups. *Cancer cell* **34**, 396-410.e398, doi:10.1016/j.ccell.2018.08.004 (2018).

11 Li, H. & Durbin, R. Fast and accurate short read alignment with Burrows–Wheeler transform. *Bioinformatics* **25**, 1754-1760, doi:10.1093/bioinformatics/btp324 (2009).

12 Li, H. *et al.* The Sequence Alignment/Map format and SAMtools. *Bioinformatics* **25**, 2078-2079, doi:10.1093/bioinformatics/btp352 (2009).

13 Van der Auwera, G. A. *et al.* From FastQ data to high confidence variant calls: the Genome Analysis Toolkit best practices pipeline. *Current protocols in bioinformatics* **43**, 11.10.11-11.10.33, doi:10.1002/0471250953.bi1110s43 (2013).

14 DePristo, M. A. *et al.* A framework for variation discovery and genotyping using next-generation DNA sequencing data. *Nat Genet* **43**, 491-498, doi:10.1038/ng.806 (2011).

15 McKenna, A. *et al.* The Genome Analysis Toolkit: a MapReduce framework for analyzing next-generation DNA sequencing data. *Genome research* **20**, 1297-1303, doi:10.1101/gr.107524.110 (2010).

16 Milani, P. *et al.* Cell freezing protocol suitable for ATAC-Seq on motor neurons derived from human induced pluripotent stem cells. *Scientific reports* **6**, 25474, doi:10.1038/srep25474 (2016).

17 Bolger, A. M., Lohse, M. & Usadel, B. Trimmomatic: a flexible trimmer for Illumina sequence data. *Bioinformatics* **30**, 2114-2120, doi:10.1093/bioinformatics/btu170 (2014).

18 Langmead, B. & Salzberg, S. L. Fast gapped-read alignment with Bowtie 2. *Nature Methods* **9**, 357, doi:10.1038/nmeth.1923

https://www.nature.com/articles/nmeth.1923#supplementary-information (2012).

19 Zhang, Y. *et al.* Model-based analysis of ChIP-Seq (MACS). *Genome biology* **9**, R137, doi:10.1186/gb-2008-9-9-r137 (2008).

20 Schmitt, Anthony D. *et al.* A Compendium of Chromatin Contact Maps Reveals Spatially Active Regions in the Human Genome. *Cell Reports* **17**, 2042-2059, doi:https://doi.org/10.1016/j.celrep.2016.10.061 (2016).

21 Rao, Suhas S. P. *et al.* A 3D Map of the Human Genome at Kilobase Resolution Reveals Principles of Chromatin Looping. *Cell* **159**, 1665-1680, doi:https://doi.org/10.1016/j.cell.2014.11.021 (2014).

22 Labun, K. *et al.* CHOPCHOP v3: expanding the CRISPR web toolbox beyond genome editing. *Nucleic acids research* **47**, W171-W174, doi:10.1093/nar/gkz365 (2019).

23 Lin, C. Y. *et al.* Active medulloblastoma enhancers reveal subgroup-specific cellular origins. *Nature* **530**, 57-62, doi:10.1038/nature16546 (2016).

24 Li, W. *et al.* Quality control, modeling, and visualization of CRISPR screens with MAGeCK-VISPR. *Genome biology* **16**, 281, doi:10.1186/s13059-015-0843-6 (2015).
25 Hsu, J. Y. *et al.* CRISPR-SURF: discovering regulatory elements by deconvolution of CRISPR tiling screen data. *Nature Methods* **15**, 992-993, doi:10.1038/s41592-018-0225-6 (2018).
26 Robinson, J. T. *et al.* Integrative genomics viewer. *Nature biotechnology* **29**, 24-26, doi:10.1038/nbt.1754 (2011).
