# Supplementary material for: Circular extrachromosomal DNA promotes tumor heterogeneity in high-risk medulloblastoma
Source: Nat Genet. 2023 Nov 9;55(12):2189–99. doi: 10.1038/s41588-023-01551-3 (PMC10703696; doi:10.1038/s41588-023-01551-3)
Supplement: Supplementary file 2 — Reporting Summary [file 41588_2023_1551_MOESM2_ESM.pdf]

nature portfolio

Corresponding author(s): Lukas Chavez
Last updated by author(s): Aug 1, 2023

Reporting Summary

Nature Portfolio wishes to improve the reproducibility of the work that we publish. This form provides structure for consistency and transparency in reporting. For further information on Nature Portfolio policies, see our Editorial Policies and the Editorial Policy Checklist.

Statistics

For all statistical analyses, confirm that the following items are present in the figure legend, table legend, main text, or Methods section.

n/a	Confirmed	
☐	☒	The exact sample size (n) for each experimental group/condition, given as a discrete number and unit of measurement
☐	☒	A statement on whether measurements were taken from distinct samples or whether the same sample was measured repeatedly
☐	☒	The statistical test(s) used AND whether they are one- or two-sided<br>*Only common tests should be described solely by name; describe more complex techniques in the Methods section.*
☐	☒	A description of all covariates tested
☐	☒	A description of any assumptions or corrections, such as tests of normality and adjustment for multiple comparisons
☐	☒	A full description of the statistical parameters including central tendency (e.g. means) or other basic estimates (e.g. regression coefficient) AND variation (e.g. standard deviation) or associated estimates of uncertainty (e.g. confidence intervals)
☐	☒	For null hypothesis testing, the test statistic (e.g. F, t, r) with confidence intervals, effect sizes, degrees of freedom and P value noted<br>*Give P values as exact values whenever suitable.*
☒	☐	For Bayesian analysis, information on the choice of priors and Markov chain Monte Carlo settings
☒	☐	For hierarchical and complex designs, identification of the appropriate level for tests and full reporting of outcomes
☐	☒	Estimates of effect sizes (e.g. Cohen's d, Pearson's r), indicating how they were calculated

Our web collection on statistics for biologists contains articles on many of the points above.

Software and code

Policy information about availability of computer code

Data collection	ICGC WGS data were downloaded using Score Client 5.0.0. sgRNAs targeting the D458 ecDNA regulome were designed using CHOPCHOP v3.
Data analysis	WGS for samples from RCH was processed using BWA v0.7.17-r118810, samtools v0.1.1911, Picard Tools v2.12.3, and GATK v3.8-1-012-14. ecDNA was identified and classified using AmpliconArchitect 1.2, CNVkit 0.9.6, AmpliconClassifier 0.4.4, and AmpliconReconstructor 1.01. Optical mapping assembly was performed using Bionano Solve 3.6. Survival analysis was performed using Lifelines 0.21.0. All other statistical tests were performed using scipy.stats 1.5.3. Multiple-hypothesis correction was performed using statsmodels 0.12.0. Visualizations were generated using circos 0.69-9, IGV desktop 2.9.2, Juicebox 1.11.08, and Seaborn 0.9.0. Genomic tracks were generated using bedtools v2.27.1, bedGraphToBigWig v4, and deeptools v3.5.1. FISH data were processed using NuSeT commit 37bcb9c and ecSeg-i commit 901ca79. Single-cell data were processed using CellRanger ARC 2.0.0, Seurat 4.0.4, DoubletFinder 2.0, PyRanges 0.0.112, ssGSEA 10.0.11, and InferCNV 1.3.3. ATAC-seq reads were trimmed using trimmomatic 0.36; quality-checked using fastqc 0.11.7; aligned using bowtie 2.3.4.3; indexed using samtools 1.10; and deduplicated using Picard Tools 2.20.8. ATAC-seq peaks were called using MACS2 2.1.2. Hi-C reads were trimmed using trimmomatic 0.39; aligned and processed using HiC-Pro 2.11.3-beta and bowtie 2.3.5; and normalized using Juicebox 1.11.08. Hi-C interactions were called using HiCCUPS 1.22.01 or FitHiC 2.0.8. qPCR data were analyzed using GraphPad Prism 9.5.2. Fingerprint analysis v1.1 is available at https://github.com/chavez-lab/fingerprint. Analysis code specific to this manuscript is available for review at https://github.com/auberginekenobi/medullo-ecdna, and https://github.com/auberginekenobi/ecdna-quant, https://github.com/auberginekenobi/rcmb56-single-cell.

For manuscripts utilizing custom algorithms or software that are central to the research but not yet described in published literature, software must be made available to editors and reviewers. We strongly encourage code deposition in a community repository (e.g. GitHub). See the Nature Portfolio guidelines for submitting code & software for further information.

Data

Policy information about availability of data

All manuscripts must include a data availability statement. This statement should provide the following information, where applicable:

- Accession codes, unique identifiers, or web links for publicly available datasets
- A description of any restrictions on data availability
- For clinical datasets or third party data, please ensure that the statement adheres to our policy

Whole genome sequencing data analyzed in this work are under controlled access, but are available from the following sources upon request:
- ICGC and Archer patient cohorts: International Cancer Genome Consortium (https://dcc.icgc.org/)
- CBTN patient cohort: Kids First Data Resource Center (https://kidsfirstdrc.org/)
- St Jude patient cohort: St Jude Cloud (https://www.stjude.cloud/)
- MB cell line and PDX models: requests for materials and manuscript correspondence should be directed to the corresponding author.
ATAC-seq, Hi-C, single cell sequencing, and pooled CRISPRi screen data will be available from NCBI Gene Expression Omnibus (GEO). ATAC-seq: [GEO accession here]. Hi-C: [GEO accession here]. scRNA+ATAC-seq: [GEO accession here]. CRISPRi: [GEO accession here]. FISH images are available at 10.6084/m9.figshare.c.6759093.

Field-specific reporting

Please select the one below that is the best fit for your research. If you are not sure, read the appropriate sections before making your selection.

☒ Life sciences ☐ Behavioural & social sciences ☐ Ecological, evolutionary & environmental sciences

For a reference copy of the document with all sections, see nature.com/documents/nr-reporting-summary-flat.pdf

Life sciences study design

All studies must disclose on these points even when the disclosure is negative.

Sample size	No method was undertaken to predetermine sample size. Because medulloblatoma is a rare disease, we accessed all data and samples available to us.
Data exclusions	Sample ICGC_MB127 was predicted to be duplicate by fingerprinting analysis and was removed. Exclusion criteria were preestablished.
Replication	No replication experiments were performed. Two biological replicates of each cell line were grown for pooled and targeted CRISPRi experiments, and variance between replicates was addressed in subsequent linear models.
Randomization	Random allocation was not relevant to our patient data because no treatment/control experiments were performed.
Blinding	Blinding was not relevant in our study since experimental validation was focused on specific tumor cell lines with limited variance e.g. only 2 cell lines available per group.

Reporting for specific materials, systems and methods

We require information from authors about some types of materials, experimental systems and methods used in many studies. Here, indicate whether each material, system or method listed is relevant to your study. If you are not sure if a list item applies to your research, read the appropriate section before selecting a response.

Materials & experimental systems

n/a	Involved in the study	
☒	☐	Antibodies
☐	☒	Eukaryotic cell lines
☒	☐	Palaeontology and archaeology
☐	☒	Animals and other organisms
☐	☒	Human research participants
☒	☐	Clinical data
☒	☐	Dual use research of concern

Methods

n/a	Involved in the study	
☒	☐	ChIP-seq
☒	☐	Flow cytometry
☒	☐	MRI-based neuroimaging

Eukaryotic cell lines

Policy information about cell lines

Cell line source(s)	Cell lines D458 and D283 were a gift from the lab of Jae Cho (OHSU). 293T cells were purchased from ATCC (Cat# CRL-3216).

Authentication	Data obtained from all cell lines were consistent with previously published knowledge of these cell lines. STR testing was performed for all samples received from external labs and matched to public STR profiles for those cells.
Mycoplasma contamination	All cell lines tested negative for mycoplasma contamination.
Commonly misidentified lines (See ICLAC register)	No commonly misidentified cell lines were used.

Animals and other organisms

Policy information about studies involving animals; ARRIVE guidelines recommended for reporting animal research

Laboratory animals	We used immunodeficient NSG mice (NOD.Cg-PrkdcscidIl2rgtm1Wjl/SzJ, The Jackson Laboratory #005557) for RCMB56 PDX intracranial implants and tumor harvests. 5 male mice between 6 and 12 weeks old were used for each experiment.
Wild animals	This study did not involve wild animals.
Field-collected samples	This study did not involve samples collected in the field.
Ethics oversight	All experiments were performed in accordance with national guidelines and regulations, and with the approval of the the Institutional Animal Care and Use Committees (IACUC) at the Sanford Burnham Prebys Medical Discovery Institute and University of California San Diego (AUF19-055 and S12123, respectively) and the UCSD Institutional Review Board (Project #171361XF).

Note that full information on the approval of the study protocol must also be provided in the manuscript.

Human research participants

Policy information about studies involving human research participants

Population characteristics	In total the WGS cohort comprised 481 medulloblastoma tumors from 468 patients (161 female, 277 male, 30 N/A; ages 0-36; see Supplementary Table 1).
Recruitment	No participants were recruited directly for this study. All human data were accessed or generated according to patient consents for general research use. Because sample metadata were compiled in part from peer-reviewed manuscripts including one specifically addressing SHH MB, the set of samples with unknown subgroup may be modestly enriched for WNT, G3, and G4 subgroups. We do not anticipate this will affect results.
Ethics oversight	Protocols were approved by Institutional Review Boards (IRB) affiliated with the University of California San Diego and Sanford Burnham Prebys Medical Discovery Institute, and Data Access Committees (DAC) from the International Cancer Genome Consortium (ICGC), St. Jude Children's Hospital, and the Children's Brain Tumor Network (CBTN).

Note that full information on the approval of the study protocol must also be provided in the manuscript.
