# Supplementary figures and images for: Circular extrachromosomal DNA promotes tumor heterogeneity in high-risk medulloblastoma
Source: Nat Genet. 2023 Nov 9;55(12):2189–99. doi: 10.1038/s41588-023-01551-3 (PMC10703696; doi:10.1038/s41588-023-01551-3)

Ladders

amp1

amp2

3.13Mb

2.7Mb

2.35Mb

1.7Mb

1.4Mb

1Mb

1A

1B

1C

1D

1E

1F

1G

2A

2B

2C

2D

2E

2F

2G

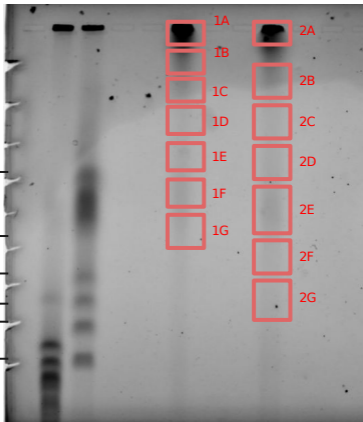

Supplement: Supplementary file 5 — Uncropped CRISPR-CATCH gel. Lanes 1 and 2 indicate CRISPR-CATCH targeting amp1 or amp2. Only amp1 was discussed in the final version. [file 41588_2023_1551_MOESM5_ESM.pdf]
